# T Cell Motility as Modulator of Interactions with Dendritic Cells

**DOI:** 10.3389/fimmu.2015.00559

**Published:** 2015-11-02

**Authors:** Jens V. Stein

**Affiliations:** ^1^Theodor Kocher Institute, University of Bern, Bern, Switzerland

**Keywords:** T cell motility, intravital imaging, thromboxane A2, T cell–DC interactions, Myo1g

## Abstract

It is well established that the balance of costimulatory and inhibitory signals during interactions with dendritic cells (DCs) determines T cell transition from a naïve to an activated or tolerant/anergic status. Although many of these molecular interactions are well reproduced in reductionist *in vitro* assays, the highly dynamic motility of naïve T cells in lymphoid tissue acts as an additional lever to fine-tune their activation threshold. T cell detachment from DCs providing suboptimal stimulation allows them to search for DCs with higher levels of stimulatory signals, while storing a transient memory of short encounters. In turn, adhesion of weakly reactive T cells to DCs presenting peptides presented on major histocompatibility complex with low affinity is prevented by lipid mediators. Finally, controlled recruitment of CD8^+^ T cells to cognate DC–CD4^+^ T cell clusters shapes memory T cell formation and the quality of the immune response. Dynamic physiological lymphocyte motility therefore constitutes a mechanism to mitigate low avidity T cell activation and to improve the search for “optimal” DCs, while contributing to peripheral tolerance induction in the absence of inflammation.

## T Lymphocytes are Cells that Scan the Surfaces of Other Cells

A defining feature of T cells is their ability to become activated by pathogen-derived peptides presented on major histocompatibility complexes (pMHCs), while remaining quiescent when self-peptides are presented. Although *in vitro* experiments have successfully delineated the molecular requirements for TCR triggering and continue to serve as important experimental tool to analyze T cell activation, there is a factor that is difficult to reproduce in reductionist *in vitro* approaches: isolated naive T cells are typically immotile *in vitro* without prior activation, whereas these cells are remarkably motile *in vivo*. Direct observation of T cell behavior using intravital two photon microscopy (2PM) has shown that these cells have a polarized, amoeboid-like shape, and move in resting lymphoid tissue with speeds of ~12–15 μm/min, in an apparently random manner (1–5). Similarly, effector T cells in non-lymphoid tissues, including leptomeningeal membranes (6), liver (7), and pancreas (8), show continuous motility, although the tissue microarchitecture is likely to restrict T cell motility, in particular, in tightly packed epithelial layers of the skin (9). The dynamic motility has evolved because T cells are MHC restricted and therefore need to physically scan the surfaces of other cells. This process is balanced with rapid decision-making on whether to arrest (e.g., to exert cytotoxic activity against a target cell) or to continue migration. A central feature of T cell biology is therefore their ability to transit from a highly motile to a stationary phenotype, i.e., from vigorous scanning to firm adhesion. Decision-making of whether to “stop” or to “go” is probably most critical in lymphoid tissue, such as peripheral lymph nodes (PLNs), where T cells move with highest speeds and are therefore only in short contact with pMHC-presenting dendritic cells (DCs). In fact, it has been estimated that each DC is contacted by ~500–5000 T cells per hour, mostly for less than a few min (10, 11). This raises two questions: first, how is the T cell motility induced in lymphoid tissue, and second, which factors influence T cell decision-making for fast arrest?

## Regulation of T Cell Motility in Lymphoid Tissue

The PLN parenchyme consists of a sponge-like network of loosely spaced fibroblastic reticular cells (FRCs) expressing the promigratory chemokines CCL19 and CCL21, ligands for the CCR7 chemokine receptor present on naïve T cells (12). DCs are closely attached to the FRC network to facilitate their continuous scanning by T cells (13). Furthermore, the LFA-1 ligands ICAM-1 and ICAM-2 are expressed on both DCs and FRCs, while CCL21 is deposited on DCs (14, 15). 2PM imaging experiments have shown that CCR7 ligands and LFA-1–ICAM-1 interactions contribute to basal migration, although even in their absence, T cells retain their amoeboid migration mode and attain considerable average speeds of ~10–12 μm/min (16–19). In line with these observations, T cells lacking the Gαi2 subunit acting downstream of CCR7 move only slightly slower than WT T cells in PLN parenchyme (20). Chemokines and integrins induce T cell polarization and the formation of a leading edge and uropod as a result of multiple signaling cascades revised elsewhere (21–24). In brief, continuous Rac-mediated F-actin assembly at the leading edge, or lamellipodium, provides the protrusion force in 3D environments, even in the absence of integrin ligands (25). This is reflected in T cells lacking the DOCK2 guanine exchange factor for the small GTPase Rac1 and 2. These cells display strongly impaired *in vitro* motility (26), and similar to Rac1/2-double-deficient T cells, show virtually no residual migration in PLN parenchyme (27, 28). Thus, LFA-1, CCR7, and other, as of yet unknown factors lead to DOCK2–Rac-driven T cell motility. The importance of this pathway for host surveillance is underscored by the recent identification of DOCK2-deficient patients, who suffer from early onset severe invasive infections (29).

Furthermore, lysophosphatidic acid (LPA) produced by the exoenzyme autotaxin (ATX) on stromal cells, including high endothelial venules (HEVs) and FRCs, contributes to transmigration and basal lymphocyte motility in PLNs (30–34). LPA binds to T cell-expressed LPA2, a member of the GPCR family, and induces Rho activation, which cooperates with CCL21 to induce contractility-dependent lymphocyte migration. Pharmacological blocking of ATX or LPA receptors or lack of LPA2 reduces T cell speeds by ~30% *in vivo*, while addition of LPA increases T cell polarization and speeds *in vitro* (30, 31, 33). These observations are in line with recent descriptions of increased cell motility generated by augmented contractility of the trailing edge in confined environments (35, 36). Similarly, T cells crossing endothelial barriers is facilitated by the Rho-GTP effector ROCK and Myosin IIA-mediated contractility to move the nucleus through narrow pores and for detachment of LFA-1–ICAM-1 adhesions (25, 37, 38). Finally, *in vitro* and *in vivo* experiments support a role for tyrosine kinase signaling downstream chemokine receptor signaling in T cells. Thus, inhibition with Janus kinases (JAK) prevents T cell chemotaxis to CCL21, adhesion to HEVs and homing (39–41). Of note, interstitial motility within lymphoid tissue was not affected by the absence of JAK1 and JAK2, pointing to compensatory mechanisms that ensure robust motility.

## Dynamic Control of T Cell Arrest

Ground-breaking work by Dustin and colleagues has uncovered that *in vitro* generated chemotactic gradients, including the prototypic T cell-attracting chemokine CCL21, are capable to disrupt TCR–pMHC complexes, leading to detachment from antigen-presenting cells and blunted T cell activation (42, 43). Although CCL21 gradients have been confirmed in interfollicular regions of PLNs (44), it remains unclear whether such gradients exist in the paracortical T cell zone or around HEVs, where naive T cells first encounter DCs (45). Interestingly, mice lacking promigratory CCR7 ligands show a delayed but ultimately enhanced T cell responses during immune responses (46). The delayed onset may result from lack of efficient T cell–DC encounters at early time points, while exceeding T cell responses at later time points are consistent with an immunosuppressive action of CCL21 via disruption of weakly reactive T cell – DC interactions.

**Figure 1:**
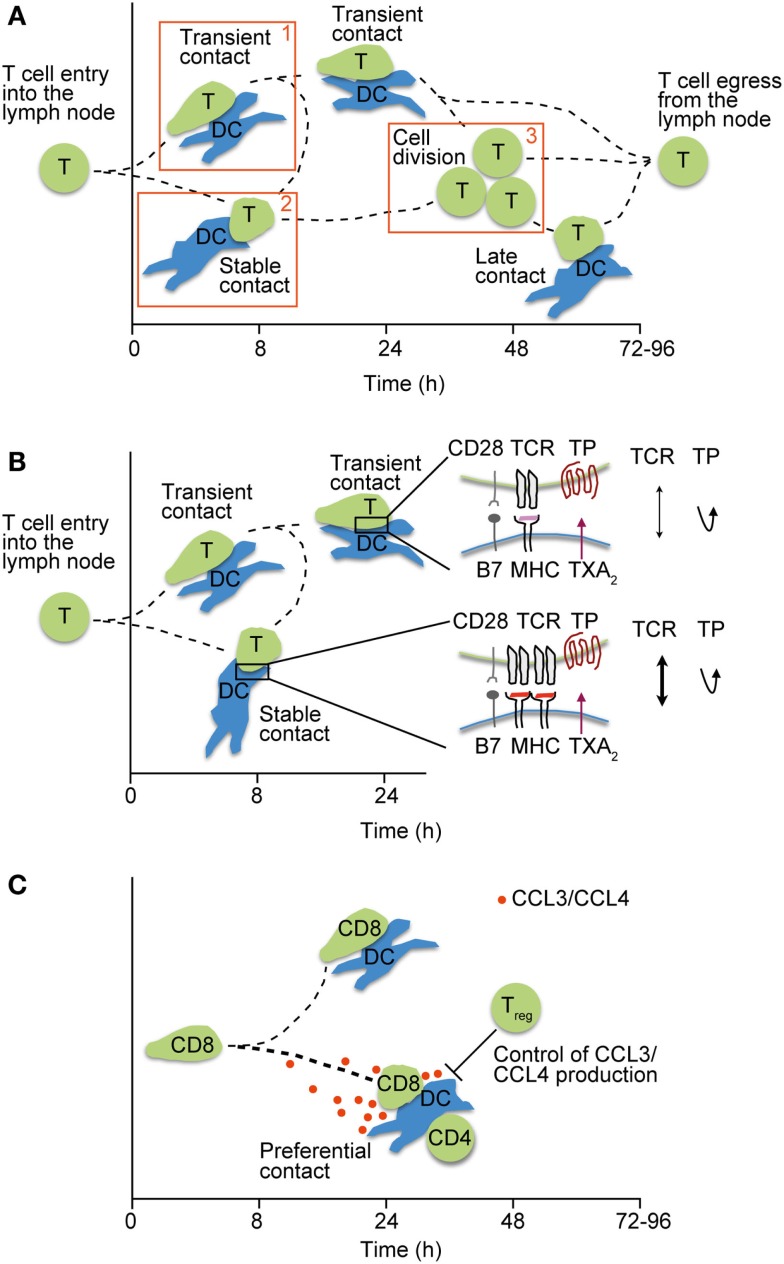
**Motile T cell–DC interactions in lymphoid tissue**. **(A)** Phases of interactions between pMHC-loaded DCs and T cells after their entry into lymph nodes. Phase 1 is characterized by transient interactions, whereas T cells engage stably with DCs during phase 2. In phase 3, T cells detach from DCs and begin to divide before egressing as effector T cells. Adapted from Ref. (55). **(B)** Regulation of weak T cell–DC interactions by TXA2 secretion. TP-induced motility prevents stable T cell attachment (phase 1–2 transition) unless a critical pMHC threshold is presented on DCs, thus ensuring a high quality of ensuing CD4^+^ T cell responses. **(C)** Secretion of CCR5 ligands CCL3 and CCL4 by pairs of interacting CD4^+^ T cells and DCs attract naïve CD8^+^ T cells and helps to foster CD4^+^ T cell help. By contrast, excessive CCR5 ligand production in the absence of Tregs deteriorates the quality of CD8^+^ T cell responses by allowing weakly interacting clones to attach to DCs.

Two photon microscopy analysis has helped to subdivide T cell–DC interactions into distinct phases that are regulated by surface levels of pMHC on DCs, as well as the TCR–pMHC affinity. Thus, high levels of cognate pMHC are able to induce immediate arrest of reactive T cells, whereas low levels result in a continuous scanning behavior of T cells (47–49). During scanning, which can last up to 8 h and is referred to as “phase 1,” T cells are able to summate signals through active NFAT and c-fos signaling (50, 51). In addition to pMHC, ICAM-1 on DCs facilitates T cell arrest (52), whereas regulatory T cells (Tregs) prevent stable interactions with DCs in this phase (53, 54). “Phase 2” stable T cell–DC interactions last for several hours and are commonly thought to be critical for full T cell activation through the formation of an immunological synapse (IS). Thus far, the precise duration of individual stable T cell–DC contacts has proven difficult to assess *in vivo*, owing to technical limitations maintaining physiological conditions and the identical field of view during intravital imaging. After ~20 h post T cell transfer, activated T cells resume motility and detach from DCs before committing to cell division, in the so-called phase 3 (Figure 1A).

Early *in vitro* evidence suggests that decision-making leading to arrest on DCs may require only a few seconds and correlates with induction of Ca flux in responsive T cells (56, 57). Interestingly, dynamic DOCK2-driven F-actin assembly at the leading edge of motile T cells is maintained during interactions with DCs, but with a different spatial arrangement at the IS interface. TCR signaling and ICAM-1–LFA-1-mediated adhesion convert Rac-driven protrusion activity at the lamellipodium of migrating T cells into an annular F-actin ring with centripetal directionality at the IS interface (58, 59). Combining these *in vitro* observations with 2PM data, the decision-making of motile T cells to undergo conversion from translocation to arrest requires a threshold pMHC level on DCs, sufficient integrated signals from previous DC encounters and LFA-1-mediated firm adhesion. Weak activatory signals because of low pMHC levels, low TCR–pMHC affinity or lack of ICAM-1 on DCs would be insufficient to trigger F-actin conversion to a ring-like structure. Consistent with this model, inhibitory receptors of TCR signaling including CTLA-4 and PD-1 prevent T cell adhesion to antigen-presenting cells (60–62).

Recent work by Krummel and colleagues has shown that naïve T cells express Myo1g, a regulator of membrane tension. When T cells are exposed to physical pressure, Myo1g accumulates at the spot of pressure exertion, followed by adjacent generation of a newly formed F-actin protrusion (63). *In vitro* and *in vivo* assays uncovered that Myo1g activity results in a meandering migration pattern, since Myo1g-deficient T cells showed less turning behavior and higher directionality. As a result, Myo1g-deficient T cells displayed shorter interaction times with DCs *in vivo*, as the average scanning time decreased from ~1.7 to 1 min. This reduced interaction time had a significant impact for T cell engagement with rare DCs, since Myo1g-deficient T cells were unable to “read” enough DC activation signals for arrest conversion and maintained their migratory behavior (63). In sum, *in vivo* migrating T cells need a minimal DC scanning time of ~100 s to integrate sufficient TCR signals for a successful conversion of protruding to annular F-actin and IS formation. This process is supported by LFA-1 adhesion to ICAM-1 on DCs.

## Thromboxane A2-Induced Motility as Quality Control for CD4^+^ T Cell Responses

Activated DCs increase surface expression of costimulatory molecules and are therefore able to efficiently activate T cells, including weakly reactive or potentially self-reactive clones. Work by Sanui and colleagues has identified activated DCs and macrophages as important source of thromboxane A2 (TXA2). TXA2 is a secreted lipid with short half-life that binds the Gα12/13-coupled TP receptor expressed on naïve but not effector/memory T cells. Binding of TXA2 to TP induces lsc (also known as p115 RhoGEF or ArhGEF1)-mediated Rho activation (64), which induces chemokinetic motility *in vitro* and disruption of T cell–DC pairs (65). In a recent study, TXA2 was shown to specifically thwart the activation of weakly reactive CD4^+^ T cell clones. After immunization or infection, Ag-specific CD4^+^ T cells that bound tetramers only weakly expanded significantly more in the absence of TP. Using 2PM imaging of reactive PLNs, the absence of TP on naïve CD4^+^ T cells resulted in increased interactions with DCs presenting low amounts or weak agonist pMHC, leading to a premature phase 1 to phase 2 transition and excessive T cell expansion (Figure 1B). By contrast, WT- and TP-deficient CD4^+^ T cells similarly engaged with DCs presenting high amounts of agonist pMHC, indicating that TXA2 preferentially prevents arrest of weakly reactive T cell clones (66). Thus, TXA2 allows T cells to maintain their search for DCs presenting high levels of cognate pMHC, rather than weak agonist or self-peptides. Indeed, TXA2-mediated quality control of early T cell–DC interactions prevented the excessive generation of follicular helper cells, which provide help to weakly reactive germinal center B cells. As consequence, lack of TXA2–TP signaling during T cell priming deteriorated the overall quality of the ensuing immune response (66).

## Tightly Regulated CCR5 Ligand Expression Controls the Quality of CD8^+^ T Cell Responses

A comparable link between the quality of adaptive immune responses and low affinity-driven interactions was made when examining how the absence of Tregs affected CD8^+^ T cell engagement with DCs. Previous work had shown that baseline production of CCR5 ligands CCL3 and CCL4 leads to the recruitment of naive CD8^+^ T cells to DC engaged in productive interactions with CD4^+^ T cells, resulting in CD4^+^ T cell help and effective CD8^+^ T cell memory formation (67). By contrast, in the absence of Tregs, DCs produced increased amounts of CCR5 ligands that facilitated excessive engagement of CD8^+^ T cells with DCs presenting low-affinity pMHC complexes as assessed by 2PM imaging (Figure 1C). This resulted in an overall lower quality of the adaptive immune responses, since the ratio of low versus high avidity clones was shifted towards the former population (68). Thus, similar to TXA2, motility-inducing agents influence T cell–DC interactions inside reactive PLNs that contain highly stimulatory DCs. It is interesting to note that in non-reactive PLNs, the absence of CCR5 ligand production and TXA2 permits unbiased scanning of resident DCs by naïve T cells, which is essential for efficient peripheral tolerance education.

## Concluding Remarks

Two photon microscopy-based investigation of T cell–DC interactions has uncovered factors that, in addition to pMHC abundance and affinity, regulate the dynamic interplay between these two cell types. Although tightly regulated T cell attraction to activated DCs promote adaptive immune responses by allowing rare cells to meet, excessive interactions with low-affinity pMHC-presenting DCs deteriorate the overall quality of clonal expansion. Furthermore, intrinsic wiring of the migratory behavior of T cells facilitates their scanning function. Thus, programmed Myo1g-induced meandering behavior permits sufficiently long interactions between T cells and DCs. In addition, continuous F-actin treadmilling during both migration and IS formation endows T cells with the capacity to quickly switch between migratory versus stationary modes. Thus, upon cessation of TCR signaling, T cells resume their motility, presumably to avoid overstimulation and to prepare for egress. These observations raise new interesting questions. For example, is the duration of phase 2-like stable interactions regulated by external chemoattractant gradients or cell-intrinsic mechanisms? Furthermore, desensitization of chemokine receptors, such as CCR5, and its impact on T cell motility patterns *in vivo* has not been investigated in detail. Such studies are relevant since CCR5 ligands attract cognate and non-cognate naïve CD8^+^ T cells, and would rapidly limit access to DCs unless CCR5 desensitization is allowing cell turnover. The continued examination of mechanisms that control T cell motility in lymphoid tissue and their impact on adaptive immune responses will remain a productive field of research in years to come.

## Conflict of Interest Statement

The author declares that the research was conducted in the absence of any commercial or financial relationships that could be construed as a potential conflict of interest.
